# Prognostic implications of systemic immune-inflammation index in patients with bone metastases from hepatocellular carcinoma treated with radiotherapy

**DOI:** 10.3389/fonc.2023.1076428

**Published:** 2023-05-12

**Authors:** Jingyao Chen, Wenhan Huang, Xiaohong Xu, Shaonan Fan, Qi Zhang, Xuan Li, Zhaochong Zeng, Jian He

**Affiliations:** ^1^ Department of Radiation Oncology, Zhongshan Hospital, Fudan University, Shanghai, China; ^2^ Jinshan Hospital Center for Tumor Diagnosis & Therapy, Jinshan Hospital, Fudan University Shanghai Medical School, Shanghai, China

**Keywords:** hepatocellular carcinoma, bone metastasis, radiotherapy, systemic immune-inflammation index, prognostic value

## Abstract

**Background:**

Previous studies have shown that systemic inflammation indicators could predict the survival outcomes of patients with malignant tumors receiving various treatments. Radiotherapy, as a crucial treatment modality, effectively alleviates discomfort in patients with bone metastasis (BM) and greatly improves the quality of life for them. This study aimed to investigate the prognostic value of systemic inflammation index in hepatocellular carcinoma (HCC) patients with BM treated with radiotherapy.

**Methods:**

We retrospectively analyzed clinical data collected from HCC patients with BM who received radiotherapy in our institution between January 2017 and December 2021. The pre-treatment neutrophil-to-lymphocyte ratio (NLR), platelet-to-lymphocyte ratio (PLR), and systemic immune-inflammation index (SII) were derived to determine their relationship with overall survival (OS) and progression-free survival (PFS), using the Kaplan-Meier survival curves. The optimal cut-off value of the systemic inflammation indicators for predicting prognosis was assessed by receiver operating characteristic (ROC) curves. Univariate and multivariate analyses were performed to ultimately evaluate the factors associated with survival.

**Results:**

The study included 239 patients with a median 14-month follow-up. The median OS was 18 months (95% confidence interval [CI] = 12.0-24.0) and the median PFS was 8.5 months (95% CI = 6.5-9.5). The optimal cut-off values for the patients were determined by ROC curve analysis as follows: SII =395.05, NLR=5.43 and PLR = 108.23. The area under the receiver operating characteristic curve values for SII, NLR and PLR in disease control prediction were 0.750, 0.665 and 0.676, respectively. Elevated systemic immune-inflammation index (SII>395.05) and higher NLR (NLR>5.43) were independently associated with poor OS and PFS. In multivariate analysis, Child-Pugh class (P = 0.038), intrahepatic tumor controlled (P = 0.019), SII (P = 0.001) and NLR (P = 0.007) were independent prognostic factors of OS and Child-Pugh class (P = 0.042), SII (P < 0.001) and NLR (P = 0.002) were independently correlated with PFS.

**Conclusion:**

NLR and SII were associated with poor prognosis in HCC patients with BM receiving radiotherapy and might be considered reliable and independent prognostic biomarkers for HCC patients with BM.

## Introduction

Hepatocellular carcinoma (HCC), one of the most common cancers worldwide, is an aggressive tumor, which is prone to extrahepatic metastasis that occurs in 25.5-38.5% of patients (1–3). In recent years, with the continuous prolongation of the survival period of liver cancer and the gradual progression of imaging diagnosis technology, the positive diagnosis rate of bone metastasis (BM) among HCC patients has increased significantly (4–8). Those patients often suffer pain, pathological fractures, spinal cord compression, hypercalcemia and other skeletal-related events (SRE), seriously damaging their quality of life (9). Thus, having a method for determining the survival in HCC patients with BM can us anticipate the development of detrimental symptoms stated above and prepare treatments preemptively to help mitigate them.

Recently, accumulating studies have confirmed that peripheral blood markers have prognostic significance in patients with malignant tumors (10–15). NLR, defined as neutrophil-to-lymphocyte count ratio, and PLR, defined as platelet-to-lymphocyte count ratio, are proved to be applicable biomarkers for patient prognostic evaluation and therapeutic decision-making (16, 17). SII is a comprehensive parameter, defined as the absolute platelet count multiplied by the neutrophil-to-lymphocyte count ratio (18, 19). The NLR, PLR, and SII are sensitive inflammatory markers in peripheral blood that can predict poor outcomes and prognosis for HCC patients who underwent surgical resection (20–22), liver transplantation (23, 24), stereotactic ablative radiation therapy (25), transarterial chemoembolization (26) or sorafenib treatment (27).

However, there are still much to learn about the prognostic potential of systemic inflammation biomarkers on HCC patients treated with palliative radiation therapy for bone metastases. Therefore, investigating the clinical significance of systemic Immune-Inflammation markers in those patients can further deepen our understanding of tumor inflammation and help us manage the wellbeing of our patients better.

In this retrospective study, our main purpose is to investigate the prognostic value of inflammatory indexes (NLR, PLR, and SII) before radiotherapy for predicting survival outcomes in HCC patients with BM.

## Materials and methods

### Patients

Patients who were diagnosed with HCC with bone metastases between January 2017 and December 2021 and received radiotherapy for bone metastases at Zhongshan Hospital, Fudan University were retrospectively identified. The eligibility criteria were as follows: (1) Clinical diagnosis or pathologically confirmed hepatocellular carcinoma and no coinciding other malignancy; (2) Computed tomography (CT), magnetic resonance imaging (MRI), or bone scan evidence of bone metastasis at the index site; (3) Child-Turcotte-Pugh (CTP) class A or B liver function; (4) over the age of 18. The exclusion criteria were as follows: (1) pregnant or lactating women; (2) combined with other serious complications; (3) with serious infection or bleeding disease; (4) using immunosuppressive or anti-inflammatory drugs before treatment; (5) incomplete or absent follow-up. Ethics approval for the use of human subjects was obtained from the research ethics committee of Zhongshan Hospital, and informed consent was obtained from each patient.

### Data collection

Demographic information and tumor variables of all patients were collected, including gender, age, Eastern Cooperative Oncology Group performance status (ECOG PS), HCC etiologic history, liver functionality, sites and number of bone metastases, other distant metastatic sites, serum ALP and AFP and blood cell counts. Among them, blood information such as platelet(P), neutrophil (N), and lymphocyte (L) were collected from reports of routine blood samples performed within one week before the radiotherapy for bone metastases.

### Treatment and follow-up

The treatments were administrated as described previously according to our institutional protocol (28). All patients underwent external beam radiotherapy with linear accelerator beam energies ranging from 6–15 megavolts (MV). Each radiation dose was administered using the ONCOR Avant-Garde Linear Accelerator (Siemens Medical Solutions, Inc. Oncology Care Systems Group). The types and modalities of radiation therapy were chosen based on the location and size of the lesions as well as the general condition of the patients. The bone metastatic lesions were scheduled the full radiation dosage at 28-60 Gy in 5-30 fractions.

All patients were assessed *via* blood examination, CT, MRI, and bone scan at 1 to 3 months after radiotherapy completion and every 3 months thereafter. Survival data was followed up by telephone and email 3-monthly until December 2021 to understand the patient’s survival status, tumor recurrence, or time to metastasis. Overall survival (OS) was defined as the time from the initiation of radiotherapy for bone metastases to death or the last follow-up, and progression-free survival (PFS) was calculated from the time from the first day of radiotherapy for bone metastases to recurrence and deterioration, death, or final follow-up.

### Statistical analysis

The SII, NLR, and PLR were calculated as follows: SII = P ×N/L, NLR = N/L, and PLR = P/L. All statistical analyses were performed using SPSS version 26.0 (IBM, Armonk, NY, USA). The continuous variables were presented as the median ± interquartile range (IQR). The categorical variables were described by numbers and percentages. Patient characteristics were examined using the χ2 test or Fisher exact test. OS and PFS were assessed with the Kaplan-Meier to analyze the survival probability, and Log–rank test was used to calculate the significance of differences. Cox proportional hazard model was applied for the univariate and multivariate analyses to calculate the hazard ratios (HRs) and 95% confidence intervals on survival outcomes. Variables with P values <0.1 in univariable analyses were selected for multivariable analyses. Receiver operating characteristic (ROC) curve analysis was performed to determine the optimal cut-off values of serum biomarkers in predicting patient survival based on the Youden index. The area under the curve (AUC) was calculated to evaluate the discriminatory power. A two-tailed P value less than 0.05 was considered statistically significant in the study.

## Results

### Patient characteristics

The clinical characteristics of all patients are shown in **Table 1**, who were diagnosed with HCC with bone metastases between January 2017 and December 2021 in Zhongshan Hospital, Fudan University (Shanghai, China). A total of 239 patients with a median age of 58 years were retrospectively identified; 89.1% were male (213/239) and 23.6% were female (26/239). Among them, 96.7% (231/239) had an ECOG PS score of 0–1, 77.8% (186/239) were positive for hepatitis B virus, and 3.8% (9/239) for hepatitis C virus, 95.8% (229/239) and 4.2% (10/239) patients were Child-Pugh class A and B, respectively. There were 20.5% (49/239) patients diagnosed with bone metastases at the same time of diagnosis of HCC. The median radiation dose was 40 Gy (IQR, 30-45 Gy), delivered in 10-20 fractions. In addition to bone metastases, 46% of patients (110/239) had other sites of distant metastases, such as lung and adrenal gland. The blood characteristics of all patients are shown in **Table 2**. The median SII, NLR, and PLR were 705.05(IQR, 298.28-783.23), 4.73 (IQR, 2.38-6.00) and 163.56 (IQR, 95.12-196.67).

**Table 1:** Patient and treatment characteristics.

Variable	n or median	% or IQR range
**Age, years**	58	50-66
Sex		
Female	26	10.9%
Male	213	89.1%
ECOG performance status		
0-1	231	96.7%
≥2	8	3.3%
Etiology		
B-viral	186	77.8%
C-viral	9	3.8%
Non-B, non-C	44	18.4%
Child-Pugh class		
A	229	95.8%
B	10	4.2%
**Recurrent HCC**	49	20.5%
Number of intrahepatic tumors		
single	130	54.4%
multiple	109	45.6%
Size of intrahepatic tumors (cm)		
≤5	112	46.9%
>5	127	53.1%
Intrahepatic control		
controlled	150	62.8%
uncontrolled	89	37.2%
Extraosseous metastases		
absent	129	54.0%
present	110	46.0%
Lymph node metastasis		
absent	148	61.9%
present	91	38.1%
Vascular tumor thrombus		
absent	134	56.1%
present	105	43.9%
Number of BMs		
single	124	51.9%
multiple	115	48.1%
**Radiation dose, Gy**	40	30-45
**Fraction number**	11	10-20
**BED10, Gy**	50.7	39.0-58.5

ECOG, Eastern Cooperative Oncology Group; HCC, hepatocellular carcinoma; BM, bone metastasis; BED10, biological effective dose calculated using α/β = 10.

**Table 2:** Blood characteristics.

Variable	n or median	% or IQR range
AFP level		
≤400 ng/mL	159	66.50%
>400 ng/mL	80	33.50%
ALP level		
≤150 U/L	149	62.30%
>150 U/L	90	37.30%
γ-GT level		
≤75 U/L	113	47.30%
>75 U/L	126	52.70%
ALT level		
≤40 U/L	147	61.50%
>40 U/L	92	38.50%
AST level		
≤40 U/L	123	51.50%
>40 U/L	116	48.50%
**HGB(g/L)**	128.69	114.50-144.50
**WBC(10^9^/L)**	10.3	3.96-7.23
**PLT(10^9^/L)**	154.45	93.5-194.00
**Neutrophil(10^9^/L)**	4.14	2.60-5.10
**Lymphocyte(10^9^/L)**	1.16	0.70-1.50
**SII**	705.05	298.28-783.23
**NLR**	4.73	2.38-6.00
**PLR**	163.56	95.12-196.67

AFP, alpha-fetoprotein; ALP, alkaline phosphatase; γ-GT, gamma-glutamyl transferase; ALT, alanine aminotransferase; AST, aspartate aminotransferase; HGB, hemoglobin; WBC, white blood cell; PLT, platelet; SII, system immune-inflammation index; NLR, neutrophil-to-lymphocyte ratio; PLR, platelet-to-lymphocyte ratio; IQR, interquartile range.

### Sites of bone metastases and number of lesions

**Figure 1:**
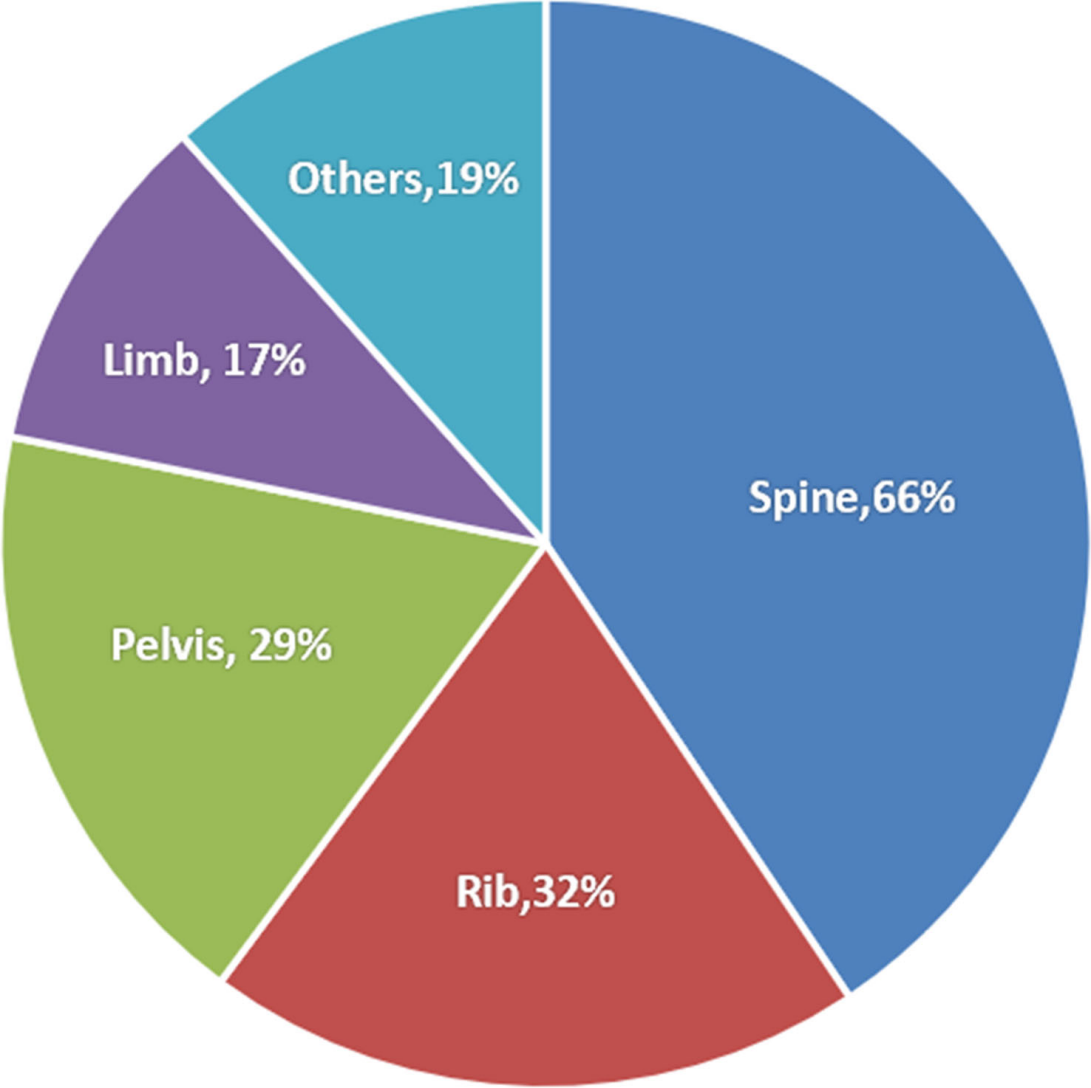
Sites of bone metastases in all patients.

In 239 patients, a total of 389 bone metastatic sites were identified. Sites of bone metastases for all patients were shown in **Figure 1**. The most common site of bone metastases was the spine (66%), followed by ribs (32%) and pelvis (29%). One hundred fifty-two patients (64%) had a single bone metastatic site, while the other patients (36%) had more than one bone lesion.

### Optimal cut-off analysis

**Figure 2:**
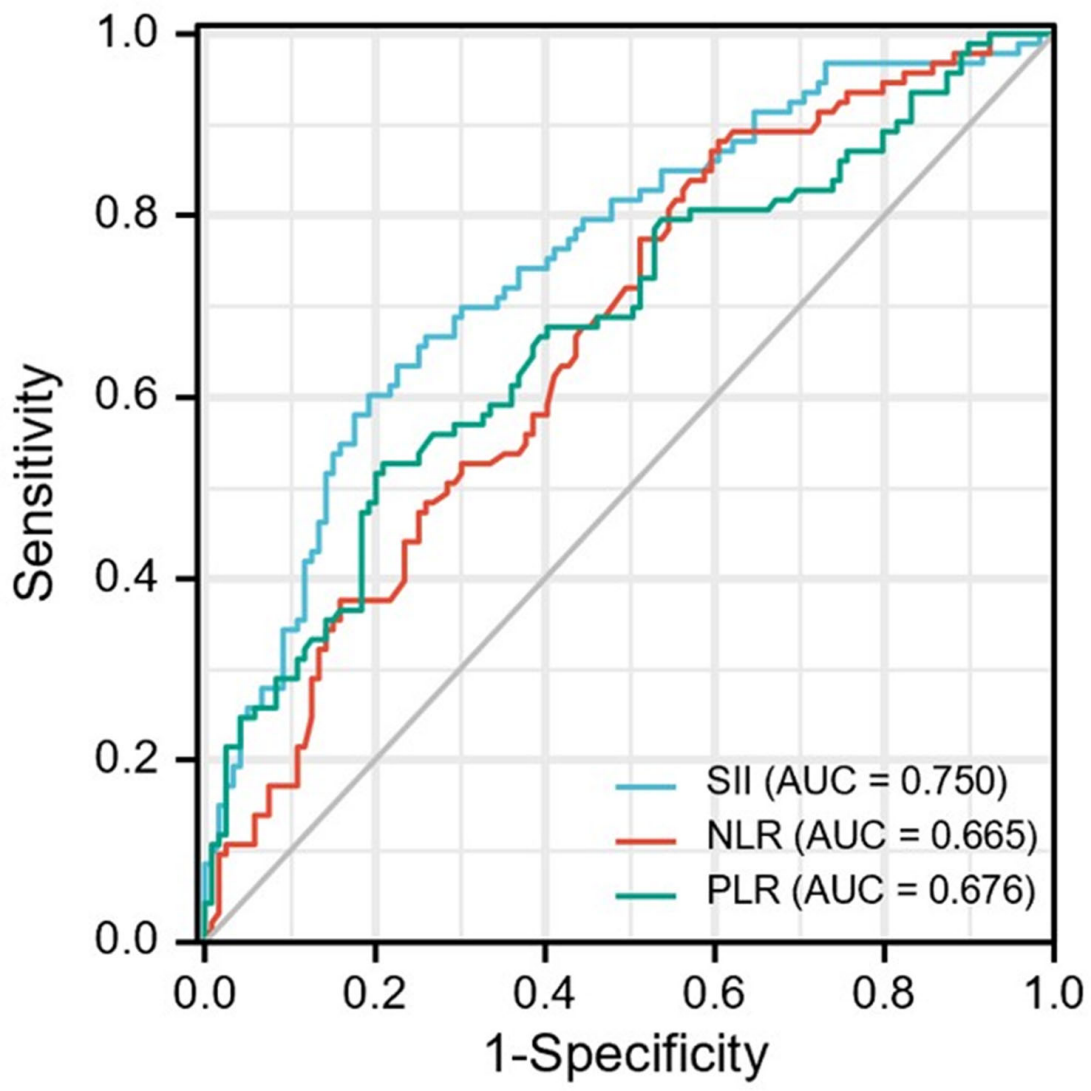
ROC curve analysis for optimal cut-off value of SII, NLR and PLR. ROC, receiver operating characteristic; SII, systemic immune-inflammation index; NLR, neutrophil-to-lymphocyte ratio; PLR, platelet-to-lymphocyte ratio.

The optimal cut-off values for the patients were determined by ROC curve analysis (**Figure 2**) as follows: SII =395.05, NLR=5.43 and PLR = 108.23. In disease control prediction, the area under the receiver operating characteristic curve values for SII, NLR, and PLR were 0.750, 0.665 and 0.676, respectively. Consequently, patients were stratified into two groups (low and high groups) based on the optimal cut-off value of each index.

### Overall survival analysis

The median follow-up duration was 14 months. The median overall survival was 18 months (95%CI, 12.0-24.0). The 1-,2-,3-year OS rate was 58.9%, 44.6%, 42.1%, respectively.

**Figure 3:**
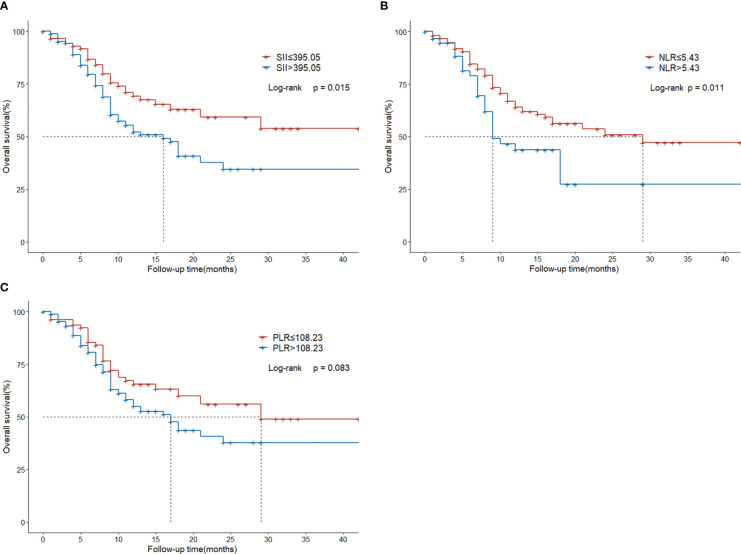
Overall survival in HCC patients with BM treated with radiotherapy based on their systemic immune-inflammation index **(A)** and neutrophil-to-lymphocyte ratio **(B)** and platelet-to-lymphocyte ratio **(C)**.

Compared with the low SII group, the high SII group had inferior survival outcomes. The median OS of the low SII group was statistically higher than that of the high SII group (NR vs. 16 months, P = 0.015; **Figure 3A**). The median OS of the low NLR group was 29 months, significantly higher than the 9 months of the high NLR group (P = 0.011; **Figure 3B**). However, there was no significant difference between low PLR and high PLR group (P=0.083; **Figure 3C**).

A total of 24 variables were applied for univariate Cox regression analysis, and P-values less than 0.1 were included in the multivariable analysis. For OS, univariate analysis indicated that ECOG performance status (P = 0.018), Child-Pugh class (P < 0.001), multiple intrahepatic tumors (P = 0.086), intrahepatic tumor controlled (P = 0.034), AFP level (P < 0.001), ALP level (P = 0.001), ALT level (P = 0.011), AST level (P = 0.007), SII (P =0.018), NLR (P = 0.014), and PLR (P = 0.092) were statistical prognostic factors. Multivariate analysis determined that Child-Pugh class (P = 0.038), intrahepatic tumor controlled (P = 0.019), SII (P = 0.001) and NLR (P = 0.007) were independent prognostic factors (**Table 3**).

**Table 3:** Univariate and multivariate cox proportional hazards analysis for overall survival.

Variable	Univariate analysis		Multivariate analysis	
	HR (95%CI)	*P* value	HR (95%CI)	*P* value
Age, years (>50)	0.950(0.492-1.833)	0.877		
Sex(male)	1.144(0.717-1.826)	0.573		
ECOG performance status≥2	3.417(1.233-9.465)	0.018	1.432(0.240-8.537)	0.693
Etiology(B-viral or C-viral)	1.071(0.624-1.838)	0.803		
Child-Pugh class(B)	5.584(2.639-11.818)	<0.001	4.659(1.090-19.910)	0.038
Recurrent HCC	0.809(0.449-1.456)	0.809		
Multiple intrahepatic tumors	1.435(0.951-2.165)	0.086	1.006(0.678-1.492)	0.978
Size of intrahepatic tumors (>5cm)	1.098(0.727-1.658)	0.656		
Intrahepatic uncontrolled	1.615(1.037-2.516)	0.034	1.651(1.088-2.504)	0.019
Extraosseous metastases	1.069(0.707-1.616)	0.751		
Lymph node metastasis	1.315(0.863-2.006)	0.203		
Vascular tumor thrombus	0.846(0.555-1.290)	0.438		
Multiple bone metastases	1.129(0.748-1.704)	0.564		
AFP level>400 ng/mL	2.619(1.705-4.022)	<0.001	1.354(0.864-2.122)	0.186
ALP level>150 U/L	2.042(1.348-3.091)	0.001	1.369(0.869-2.156)	0.175
γ-GT level>75 U/L	1.405(0.930-2.123)	0.107		
ALT level>40 U/L	1.721(1.134-2.611)	0.011	1.308(0.750-2.281)	0.344
AST level>40 U/L	1.765(1.166-2.670)	0.007	0.763(0.437-1.332)	0.341
Fraction number	1.012(0.978-1.048)	0.491		
Fraction dose, Gy	0.898(0.723-1.116)	0.333		
BED10, Gy	0.999(0.984-1.016)	0.946		
SII>395.05	1.712(1.095-2.676)	0.018	2.539(1.439-4.481)	0.001
NLR>5.43	1.780(1.121-2.825)	0.014	1.771(1.171-2.679)	0.007
PLR>108.23	1.468(0.940-2.293)	0.092	0.923(0.558-1.528)	0.755

ECOG, Eastern Cooperative Oncology Group; HCC, hepatocellular carcinoma; AFP, alpha-fetoprotein; ALP, alkaline phosphatase; γ-GT, gamma-glutamyl transferase; ALT, alanine aminotransferase; AST, aspartate aminotransferase; BED_10_, biological effective dose calculated using α/β = 10; SII, system immune-inflammation index; NLR, neutrophil-to-lymphocyte ratio; PLR, platelet-to-lymphocyte ratio.

### Progression-free survival analysis

The median progression-free survival was 8.5 months (95%CI, 6.5-9.5). The 1- and 2-year PFS rate was 36.8% and 21.2%, respectively.

**Figure 4:**
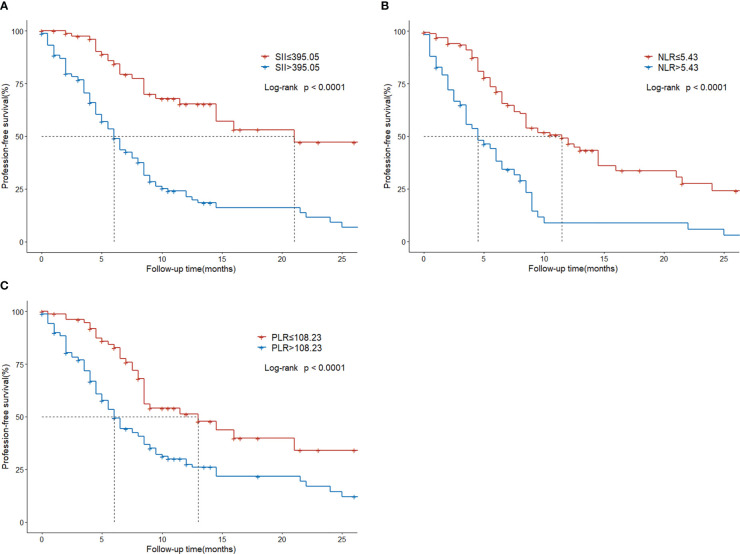
Progression-free survival in HCC patients with BM treated with radiotherapy based on their systemic immune-inflammation index **(A)** and neutrophil-to-lymphocyte ratio **(B)** and platelet-to-lymphocyte ratio **(C)**.

Regarding survival outcomes of different groups, the median PFS of the low SII group was statistically higher than that of the high SII group (21 vs. 6 months, P < 0.001; **Figure 4A**). The median PFS of the low NLR group was 11.5 months, which was significantly extended than the 4.5 months of the high NLR group (P < 0.001; **Figure 4B**). The median PFS of the low PLR group was 13 months, significantly higher than the 6.5 months of the high PLR group (P < 0.001; **Figure 4C**).

The result of the univariate analysis revealed that ECOG performance status (P = 0.052), etiology (P = 0.028), Child-Pugh class (P = 0.007), intrahepatic tumor controlled (P = 0.014), extraosseous metastases (P = 0.008), multiple bone metastases (P = 0.099), AFP level (P = 0.011), ALT level (P = 0.089), SII (P < 0.001), NLR (P < 0.001), and PLR (P < 0.001) were significant risk factors for PFS. Multivariate analysis determined that Child-Pugh class (P = 0.042), SII (P < 0.001) and NLR (P = 0.002) were independently associated with PFS (**Table 4**).

**Table 4:** Univariate and multivariate cox proportional hazards analysis for progression-free survival.

Variable	Univariate analysis		Multivariate analysis	
	HR (95%CI)	*P* value	HR (95%CI)	*P* value
Age, years (>50)	0.890(0.605-1.309)	0.555		
Sex(male)	1.008(0.567-1.792)	0.977		
ECOG performance status≥2	2.713(0.990-7.437)	0.052	2.213(0.421-11.624)	0.348
Etiology(B-viral or C-viral)	0.631(0.419-0.952)	0.028	0.995(0.653-1.517)	0.981
Child-Pugh class(B)	3.138(1.360-7.241)	0.007	4.174(1.052-16.563)	0.042
Recurrent HCC	1.402(0.920-2.136)	0.116		
Multiple intrahepatic tumors	0.924(0.649-1.316)	0.662		
Size of intrahepatic tumors (>5cm)	1.151(0.812-1.632)	0.431		
Intrahepatic uncontrolled	1.593(1.100-2.305)	0.014	1.359(0.894-2.066)	0.152
Extraosseous metastases	1.603(1.129-2.276)	0.008	1.477(0.990-2.206)	0.056
Lymph node metastasis	1.277(0.894-1.825)	0.179		
Vascular tumor thrombus	0.916(0.644-1.303)	0.627		
Multiple bone metastases	1.341(0.946-1.902)	0.099	1.217(0.814-1.818)	0.339
AFP level>400 ng/mL	1.656(1.124-2.440)	0.011	1.382(0.878-2.176)	0.162
ALP level>150 U/L	1.219(0.841-1.767)	0.295		
γ-GT level>75 U/L	1.209(0.854-1.714)	0.285		
ALT level>40 U/L	1.369(0.953-1.965)	0.089	1.067(0.695-1.637)	0.768
AST level>40 U/L	1.080(0.760-1.536)	0.667		
Fraction number	0.990(0.960-1.020)	0.497		
Fraction dose, Gy	1.095(0.936-1.281)	0.257		
BED10, Gy	1.003(0.990-1.017)	0.612		
SII>395.05	3.687(2.373-5.730)	<0.001	2.726(1.557-4.773)	<0.001
NLR>5.43	2.746(1.891-3.987)	<0.001	1.942(1.268-2.973)	0.002
PLR>108.23	2.216(1.470-3.341)	<0.001	0.897(0.544-1.478)	0.669

ECOG, Eastern Cooperative Oncology Group; HCC, hepatocellular carcinoma; AFP, alpha-fetoprotein; ALP, alkaline phosphatase; γ-GT, gamma-glutamyl transferase; ALT, alanine aminotransferase; AST, aspartate aminotransferase; BED_10_, biological effective dose calculated using α/β = 10; SII, system immune-inflammation index; NLR, neutrophil-to-lymphocyte ratio; PLR, platelet-to-lymphocyte ratio.

## Discussion

High NLR, PLR, and SII have been associated with poor survival in individuals with several solid tumors, including lung cancer, gastric cancer, colorectal cancer, and pancreatic cancer (12, 18, 29). High NLR and PLR correspond to worse OS and PFS in geriatric patients with HCC who underwent resection (29, 30). An elevated NLR and PLR independently predicted higher mortality in NSCLC patients treated with immunotherapy (11). NLR is an objective and valuable inflammatory marker that can predict survival outcomes and liver toxicity in HCC patients treated with SBRT. Likewise, post-PLR ≥263.0 was a prognostic factor of inferior PFS and OS in small hepatocellular carcinoma patients treated with SBRT (31). SII could be considered a combination of NLR and PLR and thus might be a better predictive biomarker, which has been proven to be an independent predictor in patients with HCC who received sequential therapy with sorafenib and regorafenib (32). In a meta-analysis comprising 2796 HCC patients, the results revealed that elevated pre-treatment SII was related to lower OS (HR:1.54, P < 0.001) and earlier time to recurrence (HR:1.77, P < 0.001) (33).

While previous studies have mainly focused on HCC patients receiving various other treatments, there are few reports on the prognostic role of these indicators in HCC patients with BM receiving radiotherapy. The development of bone metastasis is considered a multi-step process, including the displacement of cancer cells from the primary site, vascular invasion, distal capillary migration and attachment to bone, recruitment of inflammatory factors, and adjacent tissue invasion. Among them, systemic inflammation is an important accelerator in the proliferation, invasion, and metastasis of tumor cells. It plays an essential role in the tumor microenvironment, thus influencing cancer development and therapeutic response (34, 35). As one of the most common palliative treatments for patients with bone metastasis, radiotherapy is a crucial treatment modality, effectively alleviating discomfort and greatly improving quality of life (36). Previous studies revealed that systemic inflammation would inevitably impact radiotherapy’s efficacy (37, 38). In this study, we evaluated the association between several immune inflammatory parameters (NLR, PLR, SII) and clinical outcomes in HCC patients with BM. We demonstrated that NLR and SII were independently associated with survival outcomes in patients after radiotherapy. The optimal predictive potential of these biomarkers was determined based on the ROC curve, and the patients were divided into high- and low-value groups. Patients with NLR> 5.43 or SII >395.05 have poorer clinical outcomes. In the multivariate cox regression analyses, the results revealed that SII independently predicted OS (HR, 2.539; 95% CI, 1.439–4.481; P = 0.001) and PFS (HR, 2.726; 95% CI, 1.557-4.773; P < 0.001). NLR independently predicted OS (HR, 1.771; 95% CI, 1.171-2.679; P = 0.007) and PFS (HR, 1.942; 95% CI, 1.268-2.973; P = 0.002).

The molecular mechanisms of the prognostic significance of NLR, PLR, and SII for cancer patients may correlate with the function of platelets, neutrophils, and lymphocytes, reflecting inflammatory response and immune dysfunction. Beyond hemostasis and thrombosis, blood platelets also play a part in numerous pathways pivotal for cancer progression and metastasis. Research indicated that platelets can protect tumor cells from shear forces and assault of NK cells, and communicate with multiple growth factors, chemokines, inflammatory factors, and other immune cells, thereby inducing tumor cells proliferation and distant extravasation (39). Neutrophils, considered essential for the immune surveillance of tumor cells, exert multifaceted and sometimes opposing roles during cancer initiation, progression and dissemination (40). Neutrophils may be reprogrammed into a cancer-promoting state in the cancer microenvironment. They could produce some granule proteins (MMP-9 and ARG-1), subsequently degrading the extracellular matrix, and suppressing antigen-presentation and T lymphocyte activation, thereby resulting in immune escape, prompting cancer cells evasion and decreasing sensitivity to radiation treatment (41, 42). It is well established that as one of the most vital cells of the immune system, lymphocytes play a crucial role in tumorigenesis, cancer progression, metastatic seeding, and therapy resistance. Lymphocytes can directly interact with circulating tumor cells through the FAS-FASL axis or immune-checkpoint molecules, such as PD1-PDL1 and CTLA 4, which will induce immunosuppressive responses, leading to enhanced survival of the tumor cells (43, 44). Together with those findings, we can better understand the interactive functions of the immune inflammatory biomarkers with cancer progression and therapeutic response.

We considered several limitations to this study as follows. Firstly, this was a real-world retrospective study from only a single center, with a small sample size, and existing unavoidable in presence of objective biases. Therefore, it is reasonable to conduct additional large-scale and muti-center studies to validate the prognostic potential of immune-inflammatory indicators. Secondly, there were measurement biases because peripheral blood cell counts were performed only once. Inflammatory indicators in the peripheral blood can be influenced by infections, cirrhosis-associated hypersplenism, or medications including steroid. Lastly, HCC patients with bone metastases often sought treatment until significant symptoms had occurred, so we are unable to identify the exact number of patients with bone metastases who exhibited minor or no symptoms.

In conclusion, NLR and SII were associated with poor prognosis in HCC patients with BM receiving radiotherapy and might be considered reliable and independent prognostic biomarkers for HCC patients with BM. Furthermore, the systemic inflammation indexes are convenient and readily available during routine clinical practice, adding no additional financial burden to patients, so they are worthy of widespread use in clinical practice.

## Data availability statement

The original contributions presented in the study are included in the article. Further inquiries can be directed to the corresponding authors.

## Ethics statement

The protocol was approved by the Ethics Committee of Zhongshan Hospital, and the requirement of informed consent was waived by the Institutional Review Board since this was a retrospective analysis.

## Author contributions

JC, ZZ and JH designed the study. WH, XX and XL collected the data. JC, SF and QZ contributed to the data statistical analyses and wrote the manuscript. All authors contributed to the article and approved the submitted version.
